# Endogenous oxytocin levels in extracted saliva elevates during breastfeeding correlated with lower postpartum anxiety in primiparous mothers

**DOI:** 10.1186/s12884-022-05026-x

**Published:** 2022-09-17

**Authors:** Miyuki Nagahashi-Araki, Makoto Tasaka, Tsunehiko Takamura, Hiromi Eto, Noriko Sasaki, Wakako Fujita, Asuka Miyazaki, Kanako Morifuji, Naoko Honda, Tunetake Miyamura, Shota Nishitani

**Affiliations:** 1 Institute of Biomedical Sciences, Nagasaki University, Nagasaki, 852-8520 Japan; 2 Nagasaki Goto Chuoh Hospital, Nagasaki, Japan; 3 Department of Behavioral Medicine, National Institute of Mental Health, Tokyo, Japan; 4 Miyamura Obstetrics and Gynecology Clinic, Nagasaki, Japan; 5 Research Center for Child Mental Development, University of Fukui, Fukui, Japan; 6 Department of Child Development, United Graduate School of Child Development, Osaka University, Kanazawa University, Hamamatsu University School of Medicine, Chiba University and University of Fukui, Osaka, Japan; 7 Life Science Innovation Center, University of Fukui, Fukui, Japan

**Keywords:** Anxiety, Calming effect, Extraction, Maternity blues, Postpartum depression

## Abstract

**Background:**

Breastfeeding in the early postpartum period is expected to have mental benefits for mothers; however, the underlying psychobiological mechanisms remain unclear. Previously, we hypothesized that the release of oxytocin in response to the suckling stimuli during breastfeeding would mediate a calming effect on primiparous mothers, and we examined salivary oxytocin measurements in primiparous mothers at postpartum day 4 using saliva samples without extraction, which was erroneous. Thus, further confirmation of this hypothesis with a precise methodology was needed.

**Methods:**

We collected saliva samples at three time points (baseline, feeding, and post-feeding) to measure oxytocin in 24 primiparous mothers on postpartum day 2 (PD2) and 4 (PD4) across the breastfeeding cycle. Salivary oxytocin levels using both extracted and unextracted methods were measured and compared to determine the qualitative differences. State and trait anxiety and clinical demographics were evaluated to determine their association with oxytocin changes.

**Results:**

Breastfeeding elevated salivary oxytocin levels; however, it was not detected to a significant increase in the extraction method at PD4. We found a weak but significant positive correlation between changes in extracted and unextracted oxytocin levels during breastfeeding (feeding minus baseline); there were no other significant positive correlations. Therefore, we used the extracted measurement index for subsequent analysis. We showed that the greater the increase in oxytocin during breastfeeding, the lower the state anxiety, but not trait anxiety. Mothers who exclusively breastfed at the 1-month follow-up tended to be associated with slightly higher oxytocin change at PD2 than those who did not.

**Conclusions:**

Breastfeeding in early postpartum days could be accompanied by the frequent release of oxytocin and lower state anxiety, potentially contributing to exclusive breastfeeding.

**Supplementary Information:**

The online version contains supplementary material available at 10.1186/s12884-022-05026-x.

## Background

Breastfeeding in the early postpartum period not only promotes the recovery of the reproductive organs of the mother but also enhances the immunity of the newborn and protects against infections and allergies [1–3]. Since the physical benefits to both the mother and newborn in the early postpartum period are considerable, breastfeeding during this period has been especially promoted worldwide through the “Ten steps to successful breastfeeding” proposed by the World Health Organization (WHO) [4]. In addition, early postpartum breastfeeding has a decisive influence on the persistence of breastfeeding following the newborn period. Breastfeeding also supports the interaction and bonding between mothers and infants. Longer breastfeeding duration was associated with greater maternal-sensitive responsiveness, more attachment security, and less attachment disorganization in the child [5]. There is also a large body of evidence of the psycho-physical benefits of persistent exclusive breastfeeding, such as better attachment formation between the mother and child, which may forestall child abuse and neglect, and the benefits to the child’s intelligence and development [6, 7]. However, a few studies have examined its psychobiological basis, whereby early postpartum breastfeeding provides mental benefits to mothers.

The neuroendocrine basis of breastfeeding is driven by prolactin and oxytocin (OT), which are secreted by the pituitary gland. Prolactin is involved in milk production, and OT stimulates milk ejection by contracting the myoepithelium surrounding the breast. In recent years, OT has attracted more attention as a neurotransmitter than as a hormone for its classical properties [8]. Therefore, we hypothesized that early postpartum breastfeeding may be linked to a phenomenon that provides psychobiological benefits to mothers by specifically enhancing endogenous OT secretion and regulating the neural substrates that regulate cognition and emotion.

It has been well recognized that mothers’ plasma OT levels rise in response to the suckling stimulation during breastfeeding [9]. A study of plasma OT levels in breastfeeding mothers from 2 days to 11 weeks postpartum reported increased OT levels during breastfeeding [10, 11]. Additionally, plasma OT concentration increases within 1 min after the initiation of breastfeeding and returns to basal levels within 6 min after the end of breastfeeding, indicating that this increase in OT concentration is caused by breastfeeding [12]. However, no study has yet examined whether this endogenous OT increase by breastfeeding has a beneficial effect on the mother’s psychological state. While OT has been measured mostly using plasma, recent years have seen an expansion in the use of saliva, as salivary OT has been shown to correlate with plasma and cerebrospinal fluid OT [13–16]. However, when similar experiments were performed regarding the measurement of salivary OT, this phenomenon of increased OT concentration after breastfeeding did not always seem to be captured. For example, a study of 6-month postpartum mothers reported that salivary OT concentration during breastfeeding was highest 30 min before breastfeeding, decreased during breastfeeding, and increased slightly 30 min after the end of breastfeeding [17, 18]. Other groups have also conducted similar experiments and reported that they did not capture an increase in OT concentration [19]. In contrast, we conducted a similar experiment in first-time mothers on postpartum days 4–5, showing that salivary OT concentration increased during breastfeeding and was still higher than baseline at 30 min after breastfeeding [20]. We concluded that this might be because our experiment was conducted in the early postpartum period, unlike the other groups, and the neuroendocrine basis for the oxytocinergic system in mothers may be different. In addition, salivary OT that increased with breastfeeding was negatively correlated with maternal anxiety, indicating that endogenous OT secretion during early postpartum breastfeeding may have an anxiolytic effect [20]. However, our previous study could not eliminate the effect of false positives and had issues with the precision of the measurement because saliva OT was measured using the unextracted method. In addition, anxiety was assessed using the anxiety subscale of the shortened version of the Profile of Mood States (POMS) [21], since we did not have a specific hypothesis for the anxiolytic effect at the time. The anxiety subscale of the POMS, which presumably assesses state anxiety, is also indistinguishable from trait anxiety. In contrast, since the phase of salivary OT secretion can change at different periods after childbirth, as observed in other studies [17–19], the phase of OT secretion may differ during the first few days after childbirth. Furthermore, the symptoms of maternity blues are caused by a sudden decline in gonadal hormones after delivery, with a time lag of several days [22]. Given that the symptoms of maternity blues are transient but in some cases, worsen the symptoms of postpartum depression [22], it is valuable to compare the effects of OT on the first few days postpartum.

In the present study, to test our hypothesis, we used a more sophisticated extraction method to measure salivary OT and assessed state and trait anxiety using a standard questionnaire State-Trait Anxiety Inventory (STAI) at two time points, postpartum days 2 and 4.

## Methods

### Participants

Twenty-four Japanese mothers hospitalized in the Miyamura Obstetrics and Gynecology Clinic, Nagasaki, participated in this study on postpartum days 2 (PD2) and 4 (PD4). They were recruited during pregnancy and were eligible if they met the following criteria [20]: (i) primiparous; (ii) currently exclusively breastfeeding their infants; and (iii) had no history of mental disorders, including depression and anxiety, and no current medication use or history of psychotropic medication use. We used the following exclusion criteria: (i) recent obstetrical complications involving the mothers or their infants, (ii) delivery of a preterm infant, or (iii) delivery by cesarean section. Among the 24 mothers, two were excluded because they provided an insufficient volume of saliva. Thus, 22 mothers were included in the final analysis. The clinical demographics of the participants are presented in Table 1. These participants were independent of the participants in our previous study [20].

**Table 1:** Clinical demographics of participants

	Primiparous mothers (*n* = 22)
Age (years)	30.6 ± 5.0 (19–40)
Gestational age (week)	39.7 ± 1.1 (36.0–41.4)
Duration of labor (min)	1025.0 ± 554.6 (378–2341)
Number of nipple pores	
PD2	2.4 ± 0.8 (1–4)
PD4	5.2 ± 2.3 (1–9)
Number of breastfeeding cycles per day	
PD1	2.6 ± 1.1 (0–4)
PD2	3.7 ± 1.0 (2–6)
PD3	4.8 ± 1.1 (3–7)
Nocturnal sleep time (h)	
PD1 to 2	6.8 ± 2.1 (2.5–12)
PD3 to 4	5.5 ± 1.4 (2–8)
Breast engorgement (severe / moderate / none)	
PD2	1 (4%) / 3 (14%) / 18 (82%)
PD4	5 (23%) / 15 (68%) / 2 (9%)
Induction medicine during labor (Yes / No)	6 (27%) / 16 (73%)
Uterine contraction medicine after labor (Yes / No)	2 (9%) / 20 (91%)
Perineotomy during labor (Yes / No)	2 (9%) / 20 (91%)
Duration of breastfeeding at the experiment (min)	
PD2	11.1 ± 3.5 (5–20)
PD4	17.3 ± 3.6 (7–20)
State anxiety (STAI-State)	
PD2	39.9 ± 7.7 (27–54)
PD4	36.4 ± 9.1 (22–59)
Trait anxiety (STAI-Trait)	41.1 ± 7.3 (27–54)
Breastfeeding style at 1-month follow-up (exclusive breastfeeding / mixed or formula)	14 (64%) / 8 (36%)
Sex of child (boy / girl)	12 (45%) / 10 (55%)
Newborn crying before breastfeeding (existence /absence)	
PD2	3 (14%) / 19 (86%)
PD4	8 (36%) / 14 (64%)

Data are expressed as mean ± standard deviation. Values in parentheses are ranges or percentages

*PD* postpartum day, *STAI* State-Trait Anxiety Inventory

All participants provided written informed consent after the purpose of the experiment was explained. The experimental protocol was in accordance with the Declaration of Helsinki (2013) and was approved by the Ethics Committee of the Nagasaki University Graduate School of Biomedical Sciences.

### Procedures

As described previously [20], breastfeeding and sampling sessions were conducted by female experimenters (M.A-N and M.T) for approximately 60 min between 10:00 a.m. and 12:00 p.m. in a quiet, private, single room in the clinic. There was no music playing; the ceiling light was turned on, and the participant and experimenter were the only people in the room. Bathing for all newborns in the clinic was scheduled for 9:00 a.m. daily. We asked the participating mothers to abstain from breastfeeding after bathing and began the experiment at 10:00 a.m. in the private room. Participants did not feed the infants, nor were they swaddled during this time (9:00 a.m.–10:00 a.m.). The infant lay on a cot during the 30-min period before the experiment. Although some infants (PD2; *n* = 3/22, PD4; *n* = 8/22) were crying prior to breastfeeding (Table 1), there was no significant influence on OT when we compared OT between participants with and without infant crying using a *t*-test (*P* > 0.05). Participants were seated on comfortable chairs, and three samples of saliva were collected during the breastfeeding cycle. The first measurement was conducted 30 min before breastfeeding began (baseline), the second at the initiation of breastfeeding (+ 5 min after initiation of breastfeeding [feeding]), and the third was 30 min after the completion of breastfeeding (post-feeding). After breastfeeding was completed, most of the infants fell asleep, and the mothers placed them in the cot. We defined *ΔOT* (feeding minus baseline), which captures the OT level change in response to breastfeeding. As shown in Table 1, the third time point varied between individuals depending on the duration of breastfeeding. The experimenter did not terminate the breastfeeding session. For later correlation analyses, we calculated two indices, “Area under the curve with respect to increase” (*AUCi*) and “Area under the curve with respect to ground” (*AUCg*), which included the influence of individual duration of breastfeeding. The participants refrained from eating or drinking during the sample collection session.

### Saliva sampling and storage

Saliva samples were collected from the participants using cotton Salivettes® (Sarstedt, Rommelsdorft, Germany) at all three time points. Participants were asked to place a Salivette® in their mouth and instructed to chew for 1 min until it was saturated with saliva. The Salivettes® were kept on ice for up to 2 h, before being centrifuged at 1,500 × *g* for 15 min at 4 °C. Because of the short half-life of OT, the protease inhibitor aprotinin (500 KIU/mL, Sigma-Aldrich, St. Louis, MO, USA) was added to each collection tube to inhibit the metabolic breakdown of the peptide. The liquid samples were stored at − 80 °C.

### Salivary OT measurement by the extracted method

OT concentrations were measured using an enzyme immunoassay kit (Enzo Life Sciences, Inc.). Briefly, saliva samples (500 μL per sample) were extracted using an Oasis PRiME HLB 96-well plate at 30 mg sorbent per well (SKU: 186,008,054, Waters Inc.) and evaporated at room temperature using compressed nitrogen. Each evaporated sample was reconstituted in 125 μL of assay buffer before OT measurement to provide a sufficient sample volume (100 μL per well). This practice ensured that the plated samples contained four-fold concentrated OT, which is expected to be sufficiently high to be read above the detection limit (15.0 pg/mL). The samples were assayed using a microplate reader (SPECTRA MAX 250, Molecular Devices, LLC.) for the 96-well format according to the manufacturer’s instructions. Because each saliva sample volume was less than 1.5–2 mL, we did not measure the samples using a duplicate assay. The mean intra- and inter-assay coefficients of variation calculated by the duplicated seven standard samples were 2.1% and 6.5%, respectively.

### Salivary OT measurement by the unextracted method

To compare the differences between the extracted and unextracted method, we also measured the same samples using the unextracted method. Briefly, we used the lyophilization process instead of the extraction process to concentrate the samples four-fold overnight, reconstitute them in 125 μL of assay buffer, and store at − 20 °C until they were assayed. The detailed procedures are described in our previous manuscript [20]. The mean intra- and inter-assay coefficients of variation calculated by the duplicated seven standard samples were 3.1% and 11.1%, respectively.

### State and trait anxiety inventory (STAI)

The STAI consists of 40 self-report items related to anxiety for both state (STAI-S) and trait (STAI-T) components, adapted and standardized for the Japanese population [23]. The STAI items were rated on a 4-point Likert scale. The range for each subtest (STAI-S and STAI-T) was 20–80, with a higher score indicating greater anxiety.

### Statistical analysis

A two-way repeated-measures analysis of variance (ANOVA) was conducted to assess changes in OT levels across the within-subject factors (postpartum time point (2 [PD2 and 4]) x breastfeeding cycle (3 [baseline, feeding, and post-feeding])). Pairwise comparisons were further made with Bonferroni corrections when significant differences were observed. Additionally, one-way repeated measures ANOVA was used for easy comparison according to a previous study [20]. Significant *F* ratios were further examined using *post-hoc* Fisher’s least significant difference test. To examine whether extracted and unextracted OT were correlated, Pearson’s correlation analyses were conducted for all the OT data; each time point (baseline, feeding, and post-feeding); and *ΔOT*, *AUCi*, and *AUCg*. To examine the association between OT change indices (*ΔOT*, *AUCi*, and *AUCg*) measured by the extracted method and anxiety, Pearson’s correlation analyses between the indices and STAI (State anxiety at PD2 and 4, and Trait anxiety) were conducted with Bonferroni correction for multiple comparisons (α = 0.05/3). To demonstrate the potential relationships between OT change indices and the other demographic characteristics, all combinations of Pearson’s correlation analyses or *t*-test (two-tailed) without multiple corrections were conducted (Supplementary Figure S1). All analyses were conducted using R (ver. 4.0.5) via the RStudio platform (ver. 1.2.5019) and the rstatix software package.

## Results

### Salivary OT secretion patterns across the breastfeeding cycle measured by the extracted method

**Fig. 1:**
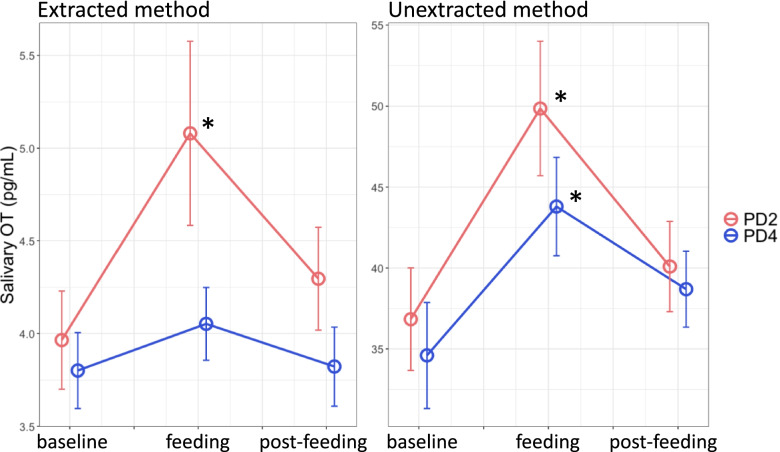
Salivary OT level changes across the breastfeeding cycle. Left: Extracted method. Right: Unextracted method. Red: PD2. Blue: PD4. *P < 0.05 (vs. baseline). Data are expressed as the mean ± standard error. PD2, postpartum day 2; PD4, postpartum day 4

OT levels at baseline, feeding, and post-feeding are shown in Fig. 1 (left). Two-way repeated-measures ANOVA revealed significant main effects for postpartum time point (PD2 and 4; [*F* (1, 21) = 4.66; *P* = 0.04, *η*_G_^2^ = 0.07]) and breastfeeding cycle (baseline, feeding, and post-feeding; [*F* (2, 42) = 4.56; *P* = 0.02, *η*_G_^2^ = 0.07]). There was no significant interaction between postpartum time point and breastfeeding cycle [*F* (2, 42) = 1.75; *P* = 0.19, *η*_G_^2^ = 0.03]. Pairwise comparisons across the breastfeeding cycle revealed that OT levels were significantly higher at feeding than at baseline (*t* = 2.52, *P*_*corr*_ = 0.047) irrespective of the postpartum time point. No other combinations of pairwise comparisons were statistically significant (baseline vs. post-feeding: *t* = 1.24, *P*_*corr*_ = 0.66; feeding vs. post-feeding: *t* = -1.87, *P*_*corr*_ = 0.21). One-way repeated-measures ANOVA for breastfeeding cycle at each postpartum time point revealed a significant difference in OT changes across the breastfeeding cycle at PD2 [*F* (2, 42) = 3.66; *P* = 0.03, *η*_G_^2^ = 0.15] but not at PD4 [*F* (2, 42) = 0.96; *P* = 0.39, *η*_G_^2^ = 0.04]. OT levels at PD2 increased significantly during feeding compared with baseline (*t* = 2.18, *P* = 0.03) and decreased between feeding and post-feeding; however, this decrease was not statistically significant (*t* = -1.53, *P* = 0.13). OT levels at post-feeding were not significantly higher than those at baseline (*t* = 0.65, *P* = 0.52).

### Salivary OT secretion patterns across the breastfeeding cycle measured by the unextracted method

OT levels at baseline, feeding, and post-feeding are shown in Fig. 1 (right). Two-way repeated-measures ANOVA revealed significant main effects for postpartum time point (PD2 and 4; [*F* (1, 21) = 3.52; *P* = 0.07, *η*_G_^2^ = 0.02]) and breastfeeding cycle (baseline, feeding, and post-feeding; [*F* (2, 42) = 10.92; *P* = 1.5e-4, *η*_G_^2^ = 0.17]). There was no significant interaction between postpartum time point and breastfeeding cycle [*F* (2, 42) = 0.45; *P* = 0.64, *η*_G_^2^ = 0.01]. Pairwise comparisons across the breastfeeding cycle revealed that OT levels were significantly higher at feeding than at baseline (*t* = 4.43, *P*_*corr*_ = 1.92e-4) and post-feeding (*t* = 2.99, *P*_*corr*_ = 0.014) irrespective of the postpartum time point. Further, no significant difference was observed between baseline and post-feeding (*t* = 1.45, *P*_*corr*_ = 0.47). One-way repeated-measures ANOVA for breastfeeding cycle at each postpartum time point revealed a significant difference in OT changes across the breastfeeding cycle at PD2 [*F* (2, 42) = 5.43; *P* = 0.008, *η*_G_^2^ = 0.21] and PD4 [*F* (2, 42) = 4.91; *P* = 0.01, *η*_G_^2^ = 0.19]. OT levels at PD2 and 4 significantly increased during feeding compared with during baseline (PD2: *t* = 2.69, *P* = 0.009, and PD4: *t* = 2.23, *P* = 0.03) and decreased between feeding and post-feeding (PD2: *t* = -2.02, *P* = 0.04); however, this decrease was not statistically significant at PD4 (*t* = -1.24, *P* = 0.22). OT levels at post-feeding were not significantly higher than those at baseline (PD2: *t* = 0.67, *P* = 0.50, and PD4: *t* = 0.99, *P* = 0.32).

### Correlations between extracted and unextracted OT

**Fig. 2:**
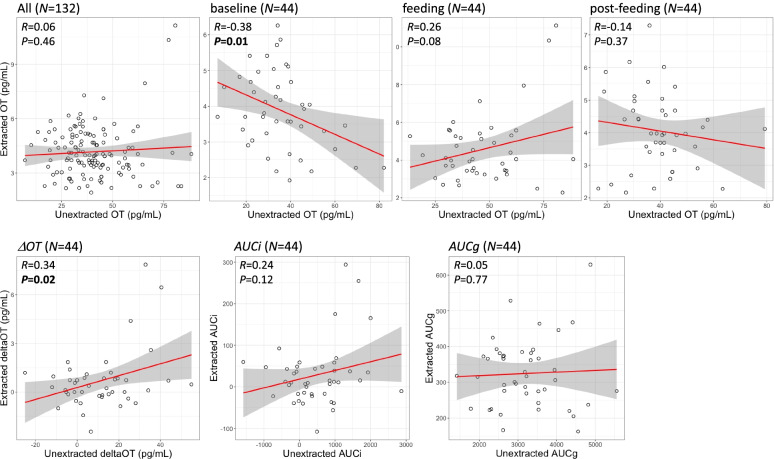
Scatter plots for the correlation analysis between the extracted and unextracted methods for OT levels and changes. Gray shade indicates 95% confidence interval

As shown in Fig. 2, significant correlations were observed between the extracted and unextracted OT at baseline (*R* = -0.38, *P* = 0.01) and *ΔOT* (*R* = 0.34, *P* = 0.02). No other pairwise correlations were significant (*P* > 0.05).

### Correlations between OT change indices measured by the extracted method and STAI

**Fig. 3:**
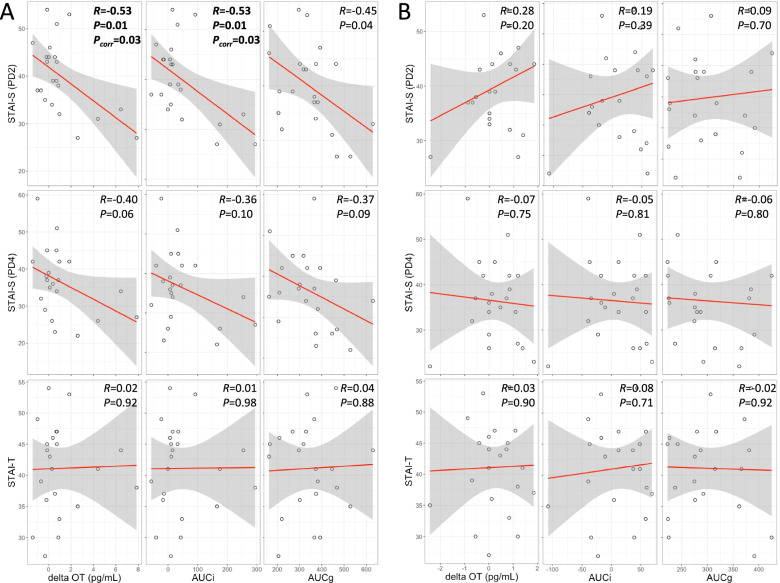
Scatter plots for the correlation analysis between changes in OT levels measured by the extracted method (A: PD2, B: PD4) and State-Trait Anxiety Inventory (STAI) scores. Gray shade indicates 95% confidence interval

We observed that the OT change indices (*ΔOT* and *AUCi*) at PD2 were significantly negatively correlated with STAI-S at PD2 (*ΔOT: R* = -0.53, *P* = 0.01, *P*_*corr*_ = 0.03 and *AUCi*: *R* = -0.53, *P* = 0.01, *P*_*corr*_ = 0.03) (Fig. 3). No other significant correlations were observed between the OT change indices and STAI.

### Correlations between OT change indices and various demographic characteristics

All the combinations of Pearson’s correlation analyses or *t*-test (two-tailed) are shown in Supplementary Figure S1. Although they were not correlated with the OT change indices at PD2, *ΔOT* and *AUCi* at PD4 were negatively correlated with “Duration of labor” (*ΔOT: R* = -0.64, *P* = 0.001, and *AUCi*: *R* = -0.72, *P* = 0.0001) and “Induction medicine during labor” (*ΔOT: R* = -0.53, *P* = 0.01, and *AUCi*: *R* = -0.59, *P* = 0.004). “Duration of labor” and “Induction medicine during labor” were also well correlated with each other (*R* = 0.67, *P* = 0.0006); as induction medicine was practically used in cases with a long duration of labor, it can be interpreted to reflect the difficulty of labor. This implies that participants with difficult labor would be less likely to show an OT increase by breastfeeding at PD4. In addition, “Induction medicine during labor” was negatively correlated with the daily number of breastfeeding cycles on PD 1 (*t* = -2.25, *P* = 0.03) and 2 (*t* = -2.13, *P* = 0.046); however, this possibility should be carefully interpreted considering that induction medicine was used in only six cases among the present study population. Although the differences were not statistically significant, participants who were exclusively breastfeeding at the 1-month postpartum follow-up (*n* = 14) had only a slightly higher trend for OT change indices at PD2 than those who were not exclusively breastfeeding (*n* = 8) (*ΔOT: t* = 1.84, *P* = 0.08, and *AUCi*: *t* = 1.69, *P* = 0.11).

## Discussion

In the present study, we investigated salivary OT changes following the breastfeeding cycle at PD2 and 4 in primiparous mothers who delivered vaginally and who experienced no abnormal symptoms such as heavy bleeding or fever during the postpartum period. We also utilized both extracted and unextracted methods for OT measurements and examined the correlation between the methods. The results showed that the extraction method showed a significant increase in OT concentration with breastfeeding. The unextracted method also showed a significant OT increase during breastfeeding. However, when we examined the correlation between the OT concentrations of the extracted and unextracted methods, we observed a correlation between the baseline values and the inverse direction, but no correlations were found at the other time points or in the total data. However, the difference between feeding and baseline (*ΔOT*) was significantly positively correlated with each other. Hence, we subsequently analyzed OT data measured via the extraction method to investigate whether increased OT associated with breastfeeding may associate with lower maternal anxiety. The results showed that the higher the OT change indices at PD2, the lower the state anxiety at PD2. Although they were not statistically significant after the multiple corrections, the OT change indices at PD2 tended to correlate significantly with state anxiety at PD4. In contrast, trait anxiety showed no correlations with those indices.

The importance of the extraction method for OT measurement has previously been raised several times [24–26]. Unlike our previous study [20] and other reports that used unextracted methods, the OT measurements in the present study were preprocessed using the extraction method. Moreover, we also measured the data using the same unextracted method, as in the previous study, and performed a novel comparison of the two conditions (the present unextracted data and population comprised a dataset different from that of our previous study [20]). Thus, we observed that the true OT concentration, which is not a false positive, is clearly different from the absolute value of the unextracted method. The correlation of the baseline data alone was significant; however, the direction of the correlation did not match. Although numerous publications [17–19, 27] have examined the relationship between baseline unextracted OT concentrations and various social and psychological metrics, we believe that these should be re-validated using extraction methods. In contrast, for *ΔOT*, there was a significant positive correlation between the extracted and unextracted methods. Although false positives are included, the amount of change may still capture the overall change associated with the true OT change, even with the unextracted method. The following discussion is based on the results obtained from the extraction method.

The present study results showed that breastfeeding increased salivary OT at PD2 but did not show a significant increase at PD4 or could not detect the changes by the saliva extraction method. However, plasma OT has been shown to capture an increase in OT even 2 months postpartum [28]. Therefore, this may be a phenomenon specific to salivary OT measurements. In particular, changes in salivary OT associated with breastfeeding appear to be influenced by the postpartum period. White-traut, et al. [18] measured salivary OT associated with the breastfeeding cycle within 8 months postpartum and found that baseline levels were the highest and decreased the most during breastfeeding. Carter, et al. (2007) [17] reported similar results. In addition, Jong, et al. (2015) [19] failed to observe changes in OT. Our PD4 results did not clearly decline, similar to those found in the studies by Carter and White-Traut; however, the results seemed to reflect a slight increase.

In the present study, for the first time, the changes in salivary OT with breastfeeding were captured using the extraction method. Although we showed that this OT increase was significantly associated with lower levels of state anxiety, it was not associated with trait anxiety. However, we cannot deny the possibility that this relationship reflects an increase in OT because the level of maternal anxiety was initially lower. Indeed, anxiety and stress influence OT secretion [29]. Unfortunately, we could not measure state anxiety as a time course change before and after breastfeeding. If this had been evaluated, causality and its sequence might have been better understood. Although we only obtained the results of each one-time assessment at PD2 and 4, we demonstrated an association between the increase in OT with breastfeeding and the lower levels of state anxiety. Because there was no association between trait anxiety and OT and the STAI was taken after the breastfeeding session, the lower maternal anxiety could be highly affected by the change in OT increase associated with breastfeeding. Reportedly, the plasma OT release during breastfeeding appears to attenuate subsequent social stress-induced cortisol increase [30].

Additionally, we examined the potential influences of other demographic characteristics that may inhibit OT secretion. A higher OT increase with breastfeeding at PD2 also showed tended to be associated with exclusive breastfeeding at the 1-month postpartum follow-up. WHO (1998) [4] encourages mothers to breastfeed for at least 6 months postpartum, recognizing physical and mental benefits to both the mother and infant. The present study results suggest that repeated self-exposure to endogenous OT triggered by breastfeeding in the immediate postpartum period in mothers could reduce their anxiety and lead to successful breastfeeding and ultimately to the health of both mother and infant. However, since our results could only indicate a potential trend, it is necessary to establish further replications. We observed an association between longer labor time and the use of induction medicine, i.e., prolonged labor and a suppressed increase in OT at PD4. In addition, when examining the relationship between the use of induction medicines and the number of breastfeeding sessions 1–3 days postpartum, the use of induction medicines was associated with fewer breastfeeding sessions. Although positive feedback from OT is essential for parturition to instigate uterine contractions, it was thought that these participants may have been vulnerable to the neurobiological mechanism of OT release. For example, DNA methylation is one of the molecular mechanisms that suppresses gene expression [31]. Hiraoka et al. [32] reported that the more the methylation in the *OXT* gene promoter region, the higher the personal distress, an aspect of affective empathy of the mothers and that there was brain gray matter volume reduction in the inferior temporal gyrus, which regulates empathy. Epigenetic mechanisms of the *OXT* gene may regulate OT secretion, leading to suppression of OT secretion during breastfeeding. Although there may have been a tendency of association with these demographics, since we did not detect a significant increase in OT during breastfeeding on PD4, these results should be cautiously interpreted, and replications would be needed with analyses attenuating false positives using multiple corrections.

The present study had four major limitations. First, the recruitment sample size was small. Since our previous study [20] had a sample size of 24, we matched that number. In addition, similar relevant studies that measured salivary OT cited in the introduction were conducted with *N* = 11 [17], *N* = 10 [18] (with some repetitions), and *N* = 4 [19]. Although the sample size of the present study may not have been sufficient for power analysis, it was larger than those of previous studies. Second, this study did not assess maternal anxiety using objective measures, such as behavioral tasks or brain MRI. In general, several studies have shown that OT has anxiolytic effects [33, 34]; however, whether self-exposure to endogenous OT caused by breastfeeding has anxiolytic effects has not been directly investigated. Although brain MRI would be difficult in the immediate postpartum period, it would have been beneficial if the anxiolytic effect could have been proven through measures, such as behavioral tasks. Third, this study was limited to primiparous women who delivered vaginally. A comprehensive study of the effects of wound pain and other factors in post-cesarean and multiparous women will be a future challenge. Finally, the follow-up on breastfeeding rates was limited to 1 month. Although we showed that high OT secretion with immediate postpartum breastfeeding was potentially associated with prolonged exclusive breastfeeding 1-month postpartum, the replications should be established, and the longer-term effects should be clarified using a prospective cohort design.

## Conclusion

Breastfeeding in the early postpartum period resulted in an accompanying increase in endogenous OT, associated with lower maternal state anxiety, and potentially contributed to the exclusive breastfeeding rate at 1-month postpartum. This suggests that early postpartum breastfeeding could be the key to successful breastfeeding, as recommended by the WHO.

## Supplementary Information

**Additional file 1:** **Supplementary Figure S1.** Pearson’scorrelation coefficients matrix.The size and color of the circles reflect correlation coefficients for eachcombination of the correlation analysis.

## Data Availability

Data cannot be shared publicly because of the restrictions of the ethics committee. Data are available upon a reasonable request to the corresponding author for researchers who meet the criteria for access to confidential data.

## Abbreviations

ANOVA: Analysis of variance
AUCg: Area under the curve with respect to ground
AUCi: Area under the curve with respect to increase
OT: Oxytocin
PD2: Postpartum day 2
PD4: Postpartum day 4
POMS: Profile of Mood States
STAI: State-Trait Anxiety Inventory
WHO: World Health Organization
