# Supplementary material for: Endogenous oxytocin levels in extracted saliva elevates during breastfeeding correlated with lower postpartum anxiety in primiparous mothers
Source: BMC Pregnancy Childbirth. 2022 Sep 17;22:711. doi: 10.1186/s12884-022-05026-x (PMC9482205; doi:10.1186/s12884-022-05026-x)
Supplement: Supplementary file 1 — Additional file 1: Supplementary Figure S1. Pearson’scorrelation coefficients matrix.The size and color of the circles reflect correlation coefficients for eachcombination of the correlation analysis. [file 12884_2022_5026_MOESM1_ESM.docx]

**Supplementary Figure S1.** Pearson’s correlation coefficients matrix. The size and color of the circles reflect correlation coefficients for each combination of the correlation analysis.
